# The “Hub Disruption Index,” a Reliable Index Sensitive to the Brain Networks Reorganization. A Study of the Contralesional Hemisphere in Stroke

**DOI:** 10.3389/fncom.2016.00084

**Published:** 2016-08-17

**Authors:** Maite Termenon, Sophie Achard, Assia Jaillard, Chantal Delon-Martin

**Affiliations:** ^1^Grenoble Institut des Neurosciences, Université Grenoble AlpesGrenoble, France; ^2^Institut National de la Santé et de la Recherche Médicale, U1216Grenoble, France; ^3^GIPSA-Lab, Université Grenoble AlpesGrenoble, France; ^4^GIPSA-Lab, Centre National de la Recherche ScientifiqueGrenoble, France; ^5^Centre Hospitalier Universitaire (CHU) de GrenobleGrenoble, France; ^6^Pole Recherche, Centre Hospitalier Universitaire (CHU) GrenobleGrenoble, France; ^7^IRMaGe, Institut National de la Santé et de la Recherche Médicale US17 Centre National de la Recherche Scientifique UMS 3552Grenoble, France

**Keywords:** graph theory, resting state fMRI, stroke, intra-hemispheric connectivity, hub disruption index, contralesional hemisphere, reliability, ICC

## Abstract

Stroke, resulting in focal structural damage, induces changes in brain function at both local and global levels. Following stroke, cerebral networks present structural, and functional reorganization to compensate for the dysfunctioning provoked by the lesion itself and its remote effects. As some recent studies underlined the role of the contralesional hemisphere during recovery, we studied its role in the reorganization of brain function of stroke patients using resting state fMRI and graph theory. We explored this reorganization using the “hub disruption index” (κ), a global index sensitive to the reorganization of nodes within the graph. For a given graph metric, κ of a subject corresponds to the slope of the linear regression model between the mean local network measures of a reference group, and the difference between that reference and the subject under study. In order to translate the use of κ in clinical context, a prerequisite to achieve meaningful results is to investigate the reliability of this index. In a preliminary part, we studied the reliability of κ by computing the intraclass correlation coefficient in a cohort of 100 subjects from the Human Connectome Project. Then, we measured intra-hemispheric κ index in the contralesional hemisphere of 20 subacute stroke patients compared to 20 age-matched healthy controls. Finally, due to the small number of patients, we tested the robustness of our results repeating the experiment 1000 times by bootstrapping on the Human Connectome Project database. Statistical analysis showed a significant reduction of κ for the contralesional hemisphere of right stroke patients compared to healthy controls. Similar results were observed for the right contralesional hemisphere of left stroke patients. We showed that κ, is more reliable than global graph metrics and more sensitive to detect differences between groups of patients as compared to healthy controls. Using new graph metrics as κ allows us to show that stroke induces a network-wide pattern of reorganization in the contralesional hemisphere whatever the side of the lesion. Graph modeling combined with measure of reorganization at the level of large-scale networks can become a useful tool in clinic.

## 1. Introduction

In numerous neurological conditions, the adult central nervous system retains an impressive capacity to recover and adapt following injury. Such so-called spontaneous recovery occurs after spinal cord injury, traumatic brain injury, and stroke. Therefore, a basic understanding of the mechanisms that underlie spontaneous recovery of function is the initial step in the development of modulatory therapies that may improve recovery rates and endpoints (Nudo, 2013). In acute stroke, it has been shown that initial damage disrupts communication in distributed brain networks. This initial disorganization is followed by a dynamic reorganization at subacute and chronic stage that may determine the level of post-stroke recovery (Carter et al., 2012). Not only disorganization in structural connectivity has been reported and related to outcome of patients (Moulton et al., 2015) but also functional reorganization in the motor network of both ipsilesional and contralesional hemispheres (Loubinoux et al., 2003; Jaillard et al., 2005; Gerloff et al., 2006; Favre et al., 2014) to compensate for the lesion itself and for remote effects (see Grefkes and Fink, 2014 for a review). The role of the contralesional hemisphere in the recovery process after stroke is supported by several studies using task fMRI paradigms (Gerloff et al., 2006; Lotze et al., 2006; Riecker et al., 2010; Rehme et al., 2011; Teki et al., 2013; Grefkes and Fink, 2014) but it has not been studied before as an independent network (without taking into account the interhemispheric connectivity) of the brain. It is thus of clinical interest to study the reorganization of the contralesional hemisphere in stroke patients by means of functional connectivity fMRI at rest.

**Figure 1:**
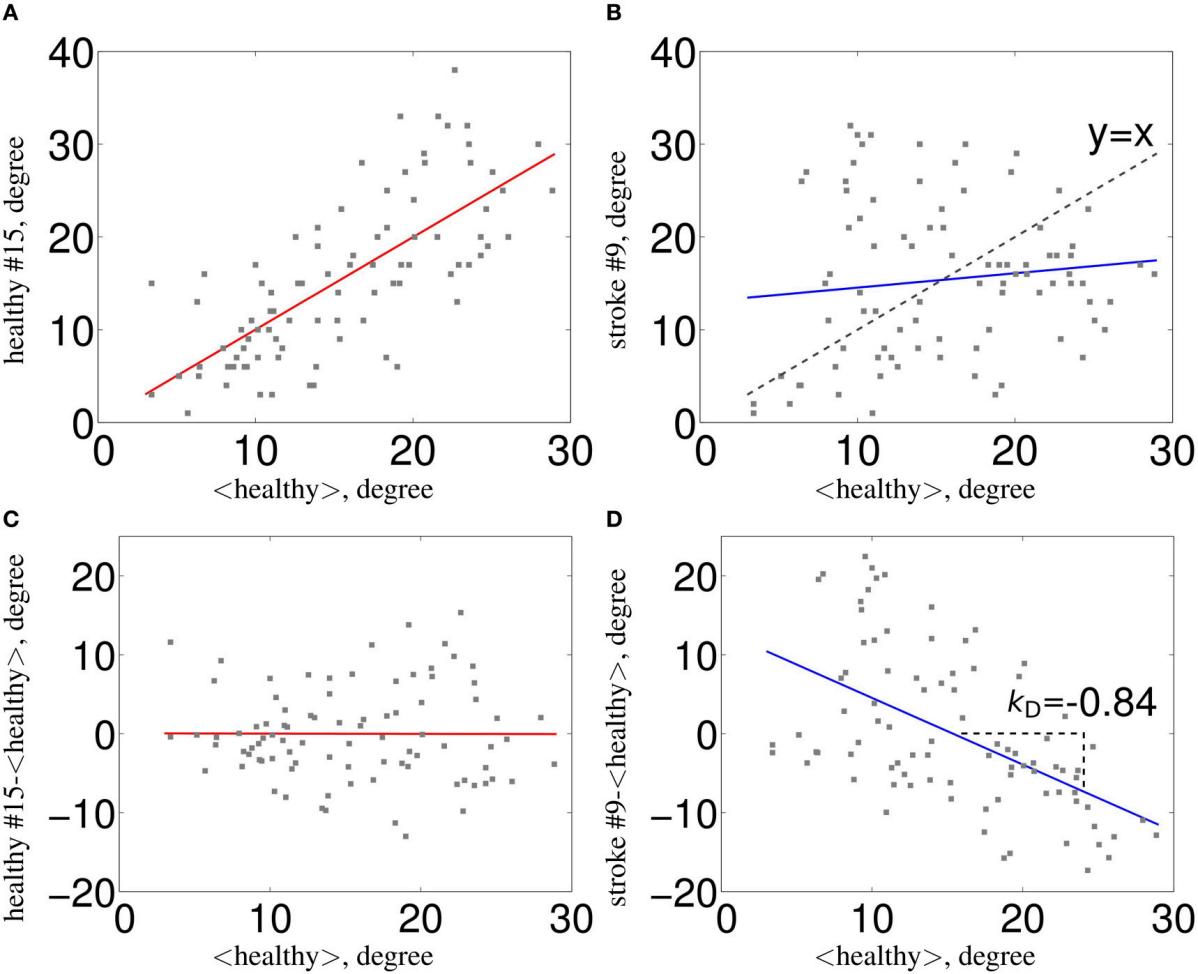
**Estimation of κ**. The nodal network topology (here, node degree) of an individual subject in relation to the normative network topology of the healthy control group **(A)** for one healthy volunteer and **(B)** for one stroke patient. To construct the hub disruption index κ for the degree, we subtract the healthy group mean nodal degree from the degree of the corresponding node in an individual subject before plotting this individual difference against the healthy group mean. κ is the slope of the regression line computed on this scatter plot. This transformation means that the data for an individual healthy volunteer **(C)** will be scattered around a horizontal line (κ~0), whereas the data for a patient in a stroke **(D)** will be scattered around a negatively sloping line (κ < 0).

In the recent years, there has been a great amount of work developing new investigation methods of the brain connectivity based on fMRI. Among those, the graph theoretical approach seems particularly useful in the context of pathology since it underlines the role of key communicating regions (hubs) in the graph. Since there was no graph metric aiming at capturing this type of reorganization after brain damage, the Hub Disruption Index (κ) was introduced in Achard et al. (2012) to capture it. κ index summarizes graph metric changes at the nodal level in a single value. It is thus a global index capturing changes at the nodal level. For a given graph metric, κ is computed as the slope of the linear regression model between the mean nodal metric value of a reference group and the differential nodal metric value between a given subject (patient or control) and that reference (see Figure 1 for a graphical explanation). If the subject's nodal values are close to those of the reference group (Figure 1C), the κ will be close to 0. Contrary, if the subject's nodal values are different from those of the reference group (Figure 1D), with reduced values in nodes with high metric values in the reference group, the κ will be negative. Once the reference group is computed, the κ can be calculated for each control and each patient individually and statistical tests can be applied to compare the differences between groups.

According to Bullmore and Sporns (2009), hubs are crucial nodes for an efficient communication in the network and are identified as nodes with high degree or high centrality values. In this paper, we computed κ using metrics that directly relate to hubs: node degree, betweenness centrality and global efficiency; and also in metrics that explore the neighborhood of the node, such as, local efficiency and clustering coefficient.

The aim of this paper is to quantify the impact of the lesion on the brain network reorganization of the contralesional hemisphere in severe stroke patients at subacute stage. For this purpose, κ index is a perfect tool to assess such reorganization by comparing nodal metrics between healthy volunteers and patients. In order to translate the use of κ in clinical context, an essential requirement to achieve meaningful results is to investigate the reliability of this index. For this purpose, we used the intraclass correlation coefficient (ICC), as it was previously assessed in several studies working with brain graphs reliability in rs-fMRI (Schwarz and McGonigle, 2011; Wang et al., 2011; Braun et al., 2012; Guo et al., 2012; Liang et al., 2012; Cao et al., 2014).

This paper is divided into three parts: in the first part, we assessed the reliability of κ, over different graph metrics, by computing the ICC in a cohort of 100 healthy subjects using the database from the Human Connectome Project (HCP)^1^. We calculated the ICCs and their *p*-values, applying bootstrap and permutation techniques to check for the influence of the number of subjects and of the number of edges (cost) in brain graphs. We also explored whether there is a laterality effect by testing the graphs of the intra-hemispheric connectivity from the left and from the right hemispheres in healthy control subjects using the HCP dataset. In the second part of the paper, we used the κ index to study the reorganization that occurs in the contralesional hemisphere of 20 severe subacute stroke patients. Finally, in the third part, we tested the robustness of the results obtained in this clinical study by randomly choosing 20 subjects as “patients” and 20 subjects as “controls” from the HCP database, computing the difference in κ between them and replicating 1000 times this procedure.

## 2. Materials and methods

### 2.1. Databases

The dataset used to assess the reliability of the κ index was selected from a large sample of rs-fMRI dataset publicly released as part of the Human Connectome Project (HCP), WU-Minn Consortium. The sample includes 100 subjects: 99 young healthy adults from 20 to 35 years old (54 females) and 1 healthy adult older than 35. Each subject underwent two rs-fMRI acquisitions on different days. Subjects were instructed to keep their eyes open and to let their mind wander while fixating a cross-hair projected on a dark background (Smith et al., 2013).

The clinical study, HERMES (PHRC2010) was the ancillary MRI study of a stem cells clinical trial, ISIS^2^. Patients were studied using fMRI at inclusion time (5 weeks post-stroke), received standard medical care, and admitted to a stroke rehabilitation center. The main inclusion criteria were: (1) right or left carotid ischemic stroke in the prior 14 days confirmed by MRI, (2) persistent moderate to severe movement deficits at one month post stroke (NIHSS > 7 and < 24), (3) optimal medical treatment (antithrombotic, antihypertensive, statins) (4) clinical status compatible with participating in the hospital rehabilitation program, and (5) willingness to participate. Patients with a previous history of neurological disease with a consequent movement deficit, claustrophobia, or psychiatric disease were excluded. Details are provided in the study website. Three out of the 31 enrolled patients were excluded: one for claustrophobia, one for refusal to continue, and one for psychiatric disease. Data from six patients were further rejected due to large motions (more than 12% of fMRI volumes rejected), and two of them had lesions in both hemispheres. Thus, the final sample comprised the 20 remaining patients, whose demographic characteristics are given in Table 1. The 20 patients were matched for age and gender with 20 healthy controls.

**Table 1:** **Demographics of stroke group**.

**Lesion**	**#**	**Age**	**Gender(M/F)**	**NIHSS**	**Lesion vol (ml)**
Right	9	49 ± 11 [27–63]	7/2	12 ± 1 [9–14]	71 ± 72 [09–241]
Left	11	56 ± 9 [38–67]	7/4	13 ± 6 [7–23]	95 ± 59 [33–220]

Mean, SD, and range for age, clinical score NIHSS and lesion volume are given.

### 2.2. Neuroimaging data acquisition

The data of Human Connectome Project were collected on the 3T Siemens Connectome Skyra MRI scanner with a 32-channel head coil. All functional images were acquired with eyes open with relaxed fixation on a projected bright cross-hair on a dark background, using a multiband gradient-echo EPI imaging sequence with the following parameters: 2 mm isotropic voxels, 72 axial slices, TR = 720 ms, TE = 33.1 ms, flip angle = 52°, field of view = 208 × 180 mm^2^, matrix size = 104 × 90, and a multiband factor of 8. 1200 images were acquired in a scan duration of 14 min and 24 s. For more detailed parameters, see (Smith et al., 2013). Two high resolution structural images T1-weighted (T1w) and T2-weighted (T2w) were further collected. They were acquired with a 3D MPRAGE sequence and a 3D T2-SPACE sequence, respectively. The main MR parameters for the T1w image were: TR = 2.4 s, TE = 2.14 ms, TI = 1000 ms, flip angle = 8°, field of view = 224 × 224 mm^2^, and 0.7 mm isotropic voxels and for the T2w: TR = 3.2 s, TE = 565 ms, flip angle = variable, field of view = 224 × 224 mm^2^, and 0.7 mm isotropic voxels; rs-fMRI data were acquired in four runs of approximately 15 min each, two runs in one session and two in another session.

In the case of the HERMES study, the MRI data of the patients and controls were acquired at a 3T (Achieva 3T TX, Philips, NL) at the IRMaGe MRI facility (Grenoble, France). The resting-state functional images were acquired using a gradient-echo EPI imaging sequence with the following parameters: in plane 3 mm isotropic voxels, 36 axial slices of 3.5 mm thick, gap = 0.25 mm TR = 2000 ms, TE = 30 ms, flip angle = 75°, field of view = 192 × 192 mm^2^. Four-hundred volumes were acquired for a total scan duration of 13 min and 20 s, with eyes open with relaxed fixation on a projected white cross-hair on a dark gray background.

### 2.3. Preprocessing pipelines

We have used two different preprocessing pipelines, one for each of the database used in this experiment.

#### 2.3.1. HCP data

T1w and T2w were corrected for bias field and distortions, coregistered together and registered to the MNI152 atlas using linear and non-linear registration functions. After registration to the atlas image, we segmented the individual T1w to obtain a gray matter (GM) probability map that was later used to extract the time series to compute the graphs. Functional data were corrected for distortions and subject motion. They were registered to the individual structural image and further to the MNI152 atlas space, using the transforms applied to the structural image. All of these preceding transforms were concatenated, together with the structural-to-MNI non-linear warp field, so that a single resulting warp (per time point) was applied to the original time series to achieve a single resampling into MNI space. Finally, the 4D image was normalized to a global mean, the bias field was removed and non-brain voxels were masked out. For more details of the preprocessing pipeline, see Glasser et al. (2013).

#### 2.3.2. HERMES study

Functional data were realigned and slice time corrected. Structural images were first coregistered to the mean EPI and segmented to obtain the GM probability map, that was elastic registered (using DARTEL Ashburner, 2007 in SPM12) onto the ICBM152 template. Resulting deformation field was then applied to the EPI and GM data to be later used to extract the time series to compute the graphs.

### 2.4. Time series extraction and graphs computation

#### 2.4.1. Time series extraction

The structural brain images were parcellated according to a modified version of the classical Anatomic-Automatic Labeling (AAL) (Tzourio-Mazoyer et al., 2002) composed of 89 regions (see Supplementary Material for more information). For the computation of the intra-hemispheric graphs, each hemisphere was divided in 44 regions, and the vermis of the cerebellum was removed from the parcellation template. Inter-hemispheric graphs were only computed to assess the reliability of κ in the whole brain. In this case, the complete parcellation scheme was used for the computation of the graphs.

In each parcel, regional mean time series were estimated by averaging, at each time point, the fMRI voxel values weighted by the GM probability of these voxels. This weighting limits the contamination of the time-series by white matter signals and cerebrospinal fluids. Residual head motion were eventually removed by regressing out motion parameters and outliers detected using the ART toolbox^3^.

#### 2.4.2. Wavelets decomposition

Wavelet transforms perform a time-scale decomposition that partitions the total energy of a signal over a set of compactly supported basis functions, each of them uniquely scaled in frequency and located in time (Achard et al., 2006). We applied the maximal overlap discrete wavelet transform (MODWT) to each regional mean time series and estimated the pairwise inter-regional correlations at each of the wavelet scales.

The wavelet decomposition is dependent on the repetition time (TR) of the rs-fMRI acquisition protocol. The databases used in this experiment have different TR. In the HCP database the TR = 0.72 s, while in the HERMES database the TR = 2.00 s, providing a maximum frequency *f* = 1/(2TR) of *f* = 0.69 Hz and *f* = 0.25 Hz, respectively. Each time a dyadic wavelet frequency band is obtained, the frequency is divided by 2. The relevant information for rs-fMRI data is then mainly contained within the scale 4, for HCP data, that represents the frequency interval 0.043−0.087 Hz, and within the scale 3, for HERMES data, that represents the frequency interval 0.032−0.065 Hz. This choice was guided by the fact that, for resting-state fMRI data, frequencies below 0.1 Hz contain the most relevant information (Biswal et al., 1995).

#### 2.4.3. Graph computation

All pairs of correlations between regions are further pooled for each of the subjects into a correlation matrix. To compute the graph, we first extracted the minimum spanning tree based on the absolute correlation matrix to keep the graph fully connected (Prim, 1957; Alexander-Bloch et al., 2010). The remaining absolute values of correlation matrices were thresholded to create an adjacency matrix that defines, for each hemisphere of each subject, an unweighted and undirected graph *G* = [*a*_*ij*_]_1 ≤ *i, j* ≤ *N*_, where *N* is the number of nodes in *G* and where *a*_*ij*_ = 0 or 1 for all the 1 ≤ *i, j* ≤ *N*. A threshold *R* was computed to produce a fixed number of edges *M*. This way the comparison between the extracted graphs is easier. Graphs are computed for different costs, which is defined as the ratio between the number of selected edges among all possible edges in the graph. More detailed information can be found in Achard et al. (2006).

In order to study the contralesional reorganization of the brain after stroke, graphs were computed for each hemisphere separately (intra-hemispheric graphs), only in the contralesional hemisphere in patients and in both, left and right hemispheres (independently) in controls. In the case of the inter-hemispheric graphs (when we assess the reliability of κ in controls), graphs were computed for the whole brain.

### 2.5. Graph metrics

Each graph metric gives a particular description of the topology of a graph. They can be computed at different levels, providing information at the global level (global metrics), about clusters inside the graph (intermediate metrics) or about each particular node (nodal metrics). Some metrics, such as local efficiency (*E*_*l*_*i*__) or clustering coefficient (*C*_*i*_), rely on the connectivity properties in the neighborhood of a node; while other metrics, such as global efficiency (*E*_*g*_*i*__) and betweenness centrality (*B*_*i*_), describe the influence of a particular node in the propagation of the information along the whole network.

The simplest graph metric is the degree of a node *i*, *D*_*i*_. It corresponds to the number of links that connect the node with the rest of nodes in the graph

$$\begin{array}{l} D_{i}=\underset{j\in N}{\sum }a_{ij}. \end{array}$$

Global efficiency measures how the information in the network is propagated. It is defined as the inverse of the harmonic mean of the minimum path length *L*_*ij*_ between a node *i* and the rest of nodes in the graph (Latora and Marchiori, 2001). It is computed as

$$\begin{array}{l} E_{g_{i}}=\frac{1}{N-1}\underset{j\in N,j\neq i}{\sum }\frac{1}{L_{ij}}. \end{array}$$

The number of shortest paths going through a node *i* is known as betweenness centrality (*B*_*i*_) (Freeman, 1977; Brandes, 2001)

$$\begin{array}{l} B_{i}=\underset{i\neq j\neq k}{\sum }\frac{\rho_{jk}\left(i\right)}{\rho_{jk}}, \end{array}$$

where ρ_*jk*_ is the number of shortest paths between nodes *j* and *k*, and ρ_*jk*_(*i*) is the number of shortest paths between nodes *j* and *k* that pass through *i*.

We have also tested the local efficiency (*E*_*l*_*i*__). It is a measure of information transfer in the immediate neighborhood of each node (Latora and Marchiori, 2001). It is computed as follows:

$$\begin{array}{l} E_{l_{i}}=\frac{1}{N_{G_{i}}\left(N_{G_{i}}-1\right)}\underset{j,k\in G_{i}}{\sum }\frac{1}{L_{jk}}, \end{array}$$

where *G*_*i*_ is a subgraph of *G* extracted from the set of nodes that are the nearest neighbours of node *i*.

Finally, we tested the clustering coefficient (*C*_*i*_) (Watts and Strogatz, 1998) which is a measure of the degree to which nodes in a graph tend to cluster together:

$$\begin{array}{l} C_{i}=\frac{1}{N}\underset{i\in N}{\sum }\frac{2t_{i}}{D_{i}\left(D_{i}-1\right)}, \end{array}$$

where *t*_*i*_ is the number of triangles around a node *i*, defined as $t_{i}=\frac{1}{2}\underset{i\neq j\neq k}{\sum }a_{ij}a_{jk}a_{ik}$ (Watts and Strogatz, 1998).

To extract the network parameters, we used *brainwaver* and *igraph* R libraries, tools that are freely available on CRAN^4^^,^^5^.

### 2.6. Hub disruption index (κ) computation

The hub disruption index (κ) was first introduced by Achard et al. (2012). It is a metric that evaluate the nodal network topology of a subject in relation to a referential network topology (i.e., the normative network topology of a healthy control group). It can be used to compare the behavior of the network of a single subject (healthy or patient) with respect to a referential network topology, but also to compare the differences between two groups with respect to a referential network topology.

Consider the case of a single healthy volunteer compared to the healthy control group (Figure 1A). Choosing one graph metric at the nodal level, for example the node degree in this figure, we plot the value of each node of the individual volunteer against the average degree for the same nodes of the healthy control group, taken as a reference. We can observe that the points fall approximately on a positive slope line of the type *y* = *x*. This means that the nodes' value of a healthy volunteer is similar to the average value of the same nodes from the group of controls. Contrary, if we proceed similarly with a stroke patient (Figure 1B), we observe that the slope line is not around the *y* = *x* line. This means that the degree of any particular node in a stroke patient is not well-predicted by the average degree of the same node in a group of healthy controls. To compute κ, we proceed as follows: we subtract the healthy group mean nodal degree (or any other nodal metric) of the same node in an individual volunteer before we plot that difference against the healthy control group mean. In this case, for a healthy volunteer (Figure 1C), the data will be scattered around an horizontal line (κ ~ 0) and for an individual patient (Figure 1D) around a negatively slope line (κ < 0).

### 2.7. Reliability of κ with intraclass correlation coefficient

The intraclass correlation coefficient (ICC) is an index that compares the variability of a metric during different sessions of the same subject to the total variation across all sessions and all subjects. It is based on the comparison of the within-subject and between-subject variability.

Following Shrout and Fleiss (1979), we applied a one-way random effect model, noted ICC (1,1), defined as:

(1) $$\begin{array}{l} ICC=\frac{s_{b}-s_{w}}{s_{b}+\left(k-1\right)s_{w}} \end{array}$$

where *s*_*b*_ is the variance between subjects, *s*_*w*_ is the variance within subjects and *k* is the number of sessions per subject. ICC is close to 1 when the reliability is high, and close to 0 when the reliability is low. It may take negative values when the variance within subjects is larger than between subjects, but this is due to statistical errors given a particular data set and should be considered as a non-reliable estimation.

### 2.8. Reliability of κ in controls

The κ index is proposed as a measure to capture network disorganization in individuals or groups as compared to a reference group. The assessment of its reliability in controls is a complementary step because if it is found as a reliable index, it will increase the chance of finding differences between groups, even if it does not assure that differences between groups will be found (Shirer et al., 2015).

We used HCP database to assess the reproducibility of κ in control subjects calculated over the five metrics explained above, for costs ranging between 10 and 75%. We used the mean of session 1 as reference to compute κ for each subject's session 1 and the mean of session 2 as reference for the κ of session 2. The between and within subjects variances were computed and ICC values for κ were derived following the formulae above. This was done for the whole group of 100 subjects and for subgroups of 20, 40, and 60 subjects applying bootstrap sampling.

For each subgroup size, to provide uncertainty and *p*-values on ICC, we randomly permuted the sessions between subjects. For that purpose, we used Simctest (Gandy, 2009). It is an open-ended sequential algorithm to compute the *p*-value of a test using Monte Carlo simulation. It guarantees that the resampling risk, the probability of a different decision than the one based on the theoretical *p*-value, is uniformly bounded by an arbitrarily small constant. A more detailed description can be found in Termenon et al. (2016).

In a complementary experiment, we also tested whether the intra-hemispheric κ index could be different between the right and the left hemispheres. Intra-hemispheric κ was computed among all the nodes of a single hemisphere. Then, we compared the intra-hemispheric κ of the left vs. the right hemisphere using as reference the mean between left and right metric values for each session of HCP database independently. We also tested across sessions the reliability of the left intra-hemispheric connectivity and of the right intra-hemispheric connectivity, separately. As reference, we used the mean between both sessions of the left hemisphere and the mean between both sessions of the right hemisphere, respectively. To evaluate the significance of these differences, statistical tests were performed using Wilcoxon rank-sum test (*p* < 0.05).

In case of no laterality effect in the intra-hemispheric connectivity in controls, we consider that the contralesional intra-hemispheric connectivity in stroke could be pooled together, independently of the side of the lesion.

### 2.9. Comparison of κ in patients and controls

Using the HERMES dataset, we studied the differences between controls and patients at different costs by computing the κ index for the five graph metrics introduced above.

We performed two types of analysis: in the first one, we pooled all the patients (*n* = 20), whatever the side of the lesion, and compared the κ of the contralesional hemisphere against the mean of left and right hemispheres in controls, without taking into account the inter-hemispheric connectivity. In the second analysis, we explored each sub-group of stroke patients according to the side of the lesion (*n* = 9 and *n* = 11 for right and left side lesions, respectively). Therefore, we compared 9 left contralesional hemispheres against 20 left hemispheres of controls and 11 right contralesional hemispheres against 20 right hemispheres of controls. In both analysis, to evaluate the significance of these differences, non-parametric tests were performed using Wilcoxon rank-sum test (*p* < 0.05).

### 2.10. Robustness of κ results in patients

For rigorous purpose, we replicated the experiment explained above on healthy subjects from the HCP database using bootstrap techniques. Due to the small number of patients and controls, 20 in each group, we wanted to check whether the statistically significant differences obtained when comparing both groups were reproducible in a group of healthy controls of the same size.

For each bootstrap iteration, we randomly selected 20 subjects which played the role of reference group and another 20 subjects that played the role of test group. As in the previous section, we performed two different analysis: in the first one, for the reference group, we computed the mean between left and right hemispheres (same way we did with the HERMES study); for the test group, we selected 9 left hemispheres and 11 right hemispheres and pooled them together in the same group. In the second analysis, we selected 11 right hemispheres (that played the role of the 11 right contralesional hemispheres of stroke patients) and compared them to 20 right hemispheres (corresponding to the 20 controls) and similarly, we selected 9 left hemispheres that were compared to 20 left hemispheres. For each of the 1000 bootstrap iterations corresponding to a selection of two groups, we computed the 5 mean graph metrics, the κ related to each metric and the *z*-value of the differences between groups.

### 2.11. Cortical surface rendering

Cortical surface representations of the distribution of the mean differences between healthy controls' and stroke patients' groups was done with Caret v5.64 software (Van Essen et al., 2001). The significance of the group differences in the above mentioned graph metrics at each region were tested using Wilcoxon test with a false positive correction *p* < (1/*N*) = 0.023 (as in Lynall et al., 2010), where *N* is the number of regions, in our case 44 in each hemisphere.

## 3. Results

### 3.1. Reliability of κ in controls

**Figure 2:**
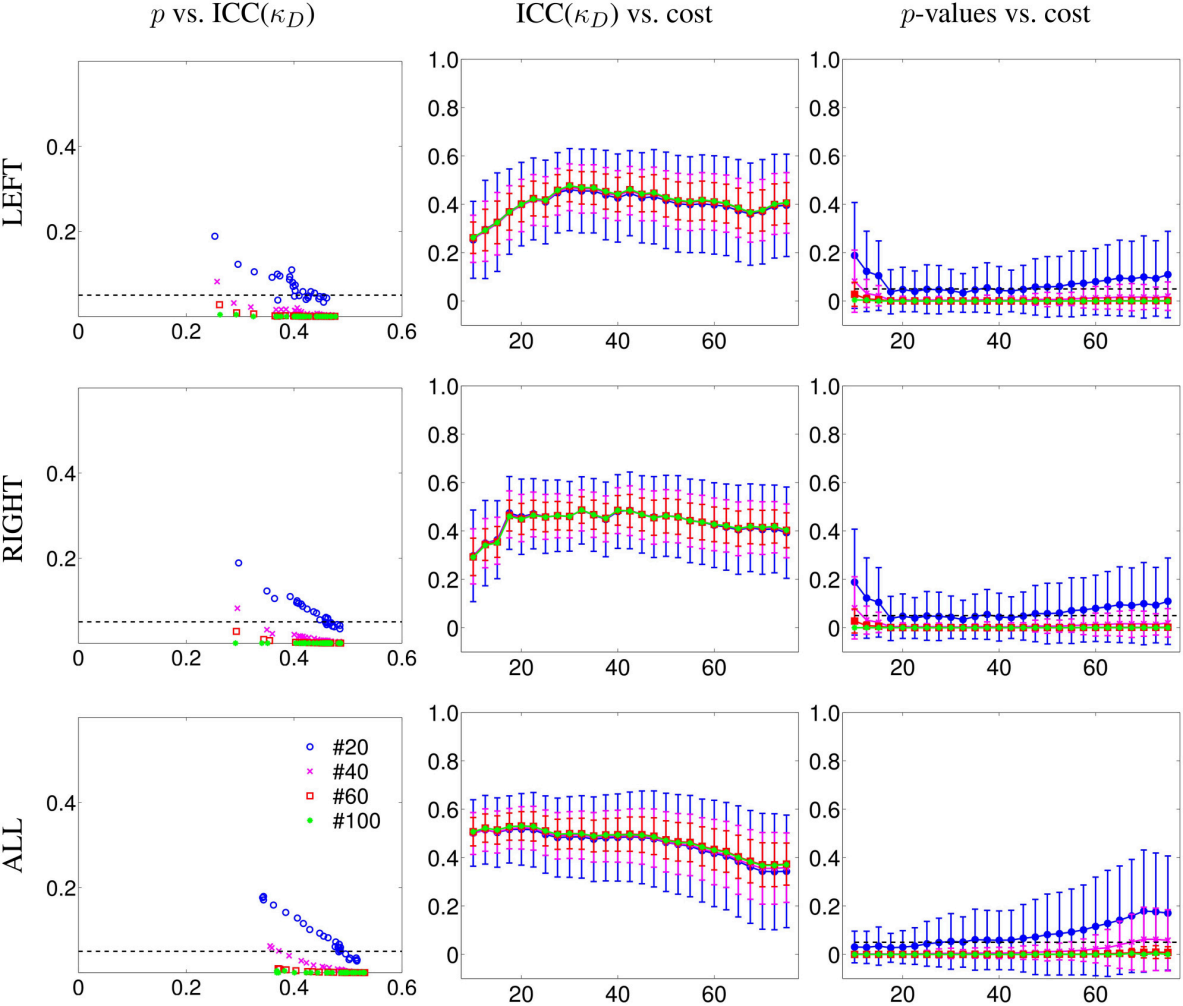
**Reliability results for κ degree (κ_*D*_) in terms of number of subjects as a function of the cost from 10 to 75%, in steps of 2.5%**. Results are given for subgroups of 20, 40, 60, and finally, 100 subjects using the database of the HCP project. First column, *p*-values of ICC (*y*-axis) as a function of ICC values (*x*-axis) for different number of subjects. Second column, values of ICC (*y*-axis) as a function of the cost (*x*-axis) for different number of subjects. Third column, ICC associated *p*-values (*y*-axis) as a function of the cost (*x*-axis) for different number of subjects. LEFT refers to the graph built from the left intra-hemispheric connections, RIGHT for the right intra-hemispheric connections. ALL refers to the graph built from connections of the whole brain. In addition, we found that κ_*D*_ is more reliable than classical metrics. We observed similar behaviors with other metrics (compare Figure S1 and Figure S2 in Supplementary Material).

To test the reliability of κ, we computed the ICC on subgroups of 20, 40, 60, and 100 subjects. We applied permutations and bootstrap techniques to assess the *p*-values of the obtained ICC. In Figure 2, we show the ICCs and their *p*-values of κ_*D*_ with respect to cost, for the different subgroups' sizes. We considered separately the left intra-hemispheric connectivity (LEFT), the right intra-hemispheric connectivity (RIGHT) and the whole brain connectivity including both intra- and inter-hemispheric connections (ALL).

In the case of intra-hemispheric connectivity, for a cost equal to or above 20%, we observed an ICC value that is roughly independent of the cost, with an uncertainty on the ICC that depends on the number of subjects (it is reduced with an increasing number of subjects). Below 17.5% cost, the graph is too sparse and the κ_*D*_ index was not reliable. When considering connections from all the brain, we achieved higher reliability than when considering only intra-hemispheric connections for costs below 40%. When the cost is high, it means that the graph is highly connected, and thus the between and within variance differences can be reduced. We have to underline here that these ICC values are also dependent on the acquisition duration, as was shown in Birn et al. (2013); Termenon et al. (2016).

Similar results were found with the other graph metrics we tested: global efficiency (Figure S1 in Supplementary Material), betweenness centrality, clustering, and local efficiency. For the sake of comparison, under the same experimental conditions (same database, graph methodology, permutation, and bootstrap sampling), the ICC(*E*_*g*_) was lower considering the whole brain (ranging between 0.30 at 20% cost and 0.40 at 40% cost) and also, the intra-hemispheric connectivity (Figure S2 in Supplementary Material). These results show that κ_*E*_*g*__ is more reliable than the average *E*_*g*_ metric obtained by averaging all the nodes.

#### 3.1.1. Comparison of κ per hemisphere in controls

The differences between left and right intra-hemispheric connections using the 100 subjects of the HCP are shown in Figure S3 of Supplementary Material. We compared the intra-hemispheric connectivity of left and right hemispheres for each session independently (upper row). To compute the κ_*D*_, we used the mean between left and right hemispheres as reference, considering only the intra-hemispheric connections (inter-hemispheric connections were excluded). We found no significant differences between the left and the right intra-hemispheric connectivity, neither in the first session nor in the second session.

In the second comparison (lower row of Figure S3 in Supplementary Material), we studied if there were differences across sessions for each hemisphere, independently. For each session, we compared the intra-hemispheric connectivity of the left hemisphere, using as reference the mean between sessions of the left intra-hemispheric connectivity. Same procedure was applied for the right hemisphere. We found no effect of the sessions on the intra-hemispheric connectivity, nor for the left hemisphere neither for the right hemisphere.

The lack of laterality effect in the intra-hemispheric connectivity supports the view that contralesional intra-hemispheric connectivity, independently of the hemispheric location of the stroke lesion, could be pooled together. This motivates the fact that, in the study on stroke patients, we pooled together the data of patients with right-sided and left-sided lesions. We also eventually performed the statistics in separated sub-groups.

Similar results were obtained with the other tested graph metrics: κ_*E*_*g*__, κ_*E*_*l*__, κ_*B*_ and κ_*C*_.

### 3.2. Hub disruption index κ in patients

**Figure 3:**
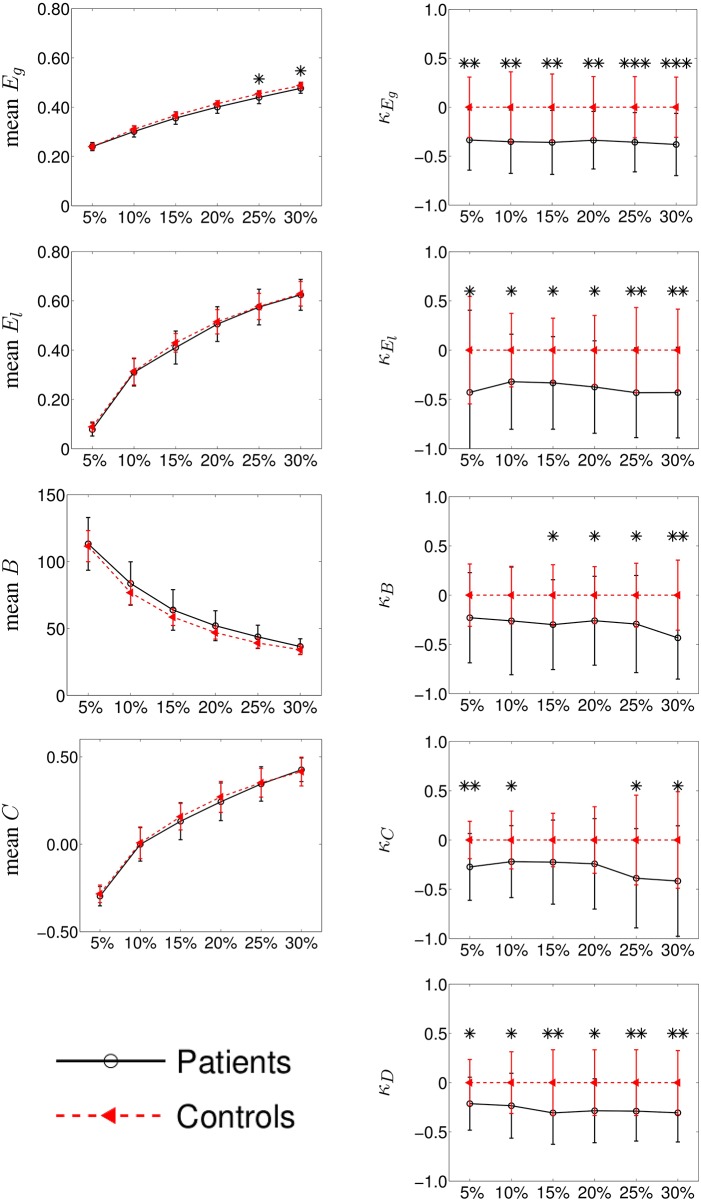
**Group differences between mean intra-hemispheric connectivity in controls and contralesional hemispheric connectivity in stroke patients according to classical graph metrics (left column) and κ index (right column)**. Metrics (*y*-axis) correspond to global efficiency (mean *E*_*g*_ and κ_*E*_*g*__, respectively), local efficiency (*E*_*l*_ and κ_*E*_*l*__), betweenness centrality (*B* and κ_*B*_), clustering coefficient (*C*, and κ_*C*_) and node degree (κ_*D*_). Cost (*x*-axis) ranges from 5 to 30%. Error bars indicate standard deviation and significant differences (Wilcoxon, *p* < 0.05) are indicated with asterisk (^*^) (^*^ < 0.05; ^**^ < 0.01; ^***^ < 0.001). Using κ, we found huge significant differences between the two groups while with classical graph metrics, differences were difficult to observe.

**Figure 4:**
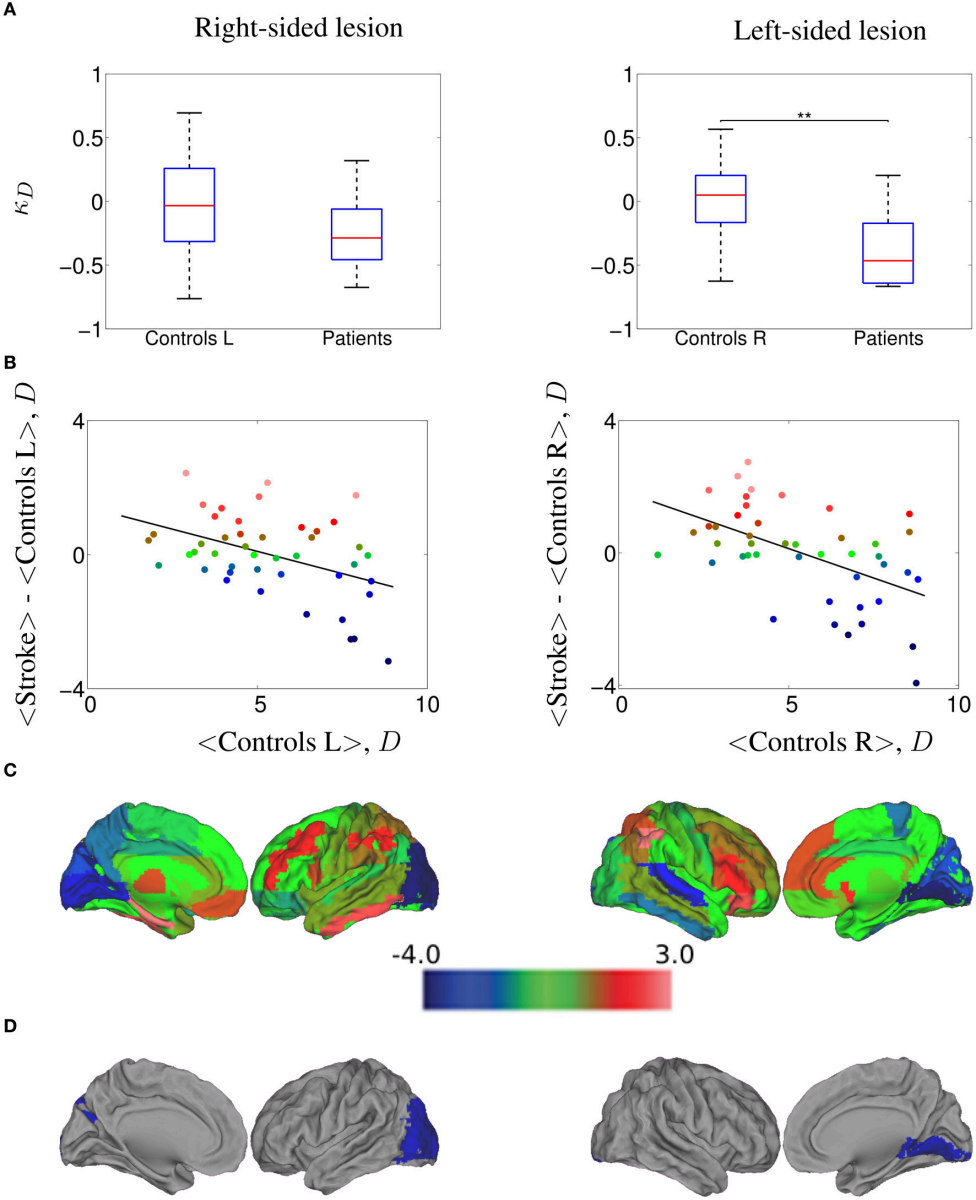
**κ_*D*_ hub disruption of functional networks in stroke patients contralesional hemisphere, computed at a 20.0% cost**. **(A)** Boxplots of the individually estimated hub disruption indices for the healthy volunteer group and the stroke patient group. On the left, healthy volunteer group left hemisphere and stroke contralesional left hemisphere; on the right, healthy volunteer group right hemisphere and stroke contralesional right hemisphere. Significant differences (Wilcoxon, *p* < 0.05) are indicated with asterisk (^*^) (^*^ < 0.05; ^**^ < 0.01; ^***^ < 0.001). **(B)** On the left, results of the healthy volunteer group left hemisphere and the stroke group with left contralesional hemisphere, where κ = −0.27; on the right, results of the healthy volunteer group right hemisphere and the stroke group with right contralesional hemisphere, where κ = −0.36. **(C)** Cortical surface representation of the difference in mean *D* between both groups; red denotes increased *D*, on average, in patients compared with healthy volunteers; blue denotes abnormally decreased *D* in stroke patients. **(D)** nodes that demonstrated significant between-group difference in nodal *D*; Wilcoxon test, *p* < 0.023; red denotes significantly increased *D* and blue denotes significantly decreased *D* in the patients on average.

We performed two different experiments. First, as we did not find any significant difference between left and right hemispheres in controls, we pooled the left and right contralesional hemispheres of stroke patients into a single group and compared them against the mean between left and right hemispheres of the controls. The results for graph metrics and κ are shown in Figure 3. Second, we analyzed each hemisphere independently in patients and controls. Results are shown in Figure 4 for κ_*D*_ and in Figures S4–S7 of the Supplementary Material for the other tested graph metrics.

#### 3.2.1. κ in patients vs. controls

In the right column of Figure 3, we show the comparison among the κ values of *E*_*g*_, *E*_*l*_, *B*, *C*, and *D* between the mean controls' left and right intra-hemispheric connectivity and patients' contralesional hemispheric connectivity, for costs ranging between 5 and 30%. We found that κ index was significantly reduced in patients as compared to controls for κ_*E*_*g*__, κ_*E*_*l*__ and κ_*D*_ at all costs and for κ_*B*_ at costs above 10%. In the case of κ_*C*_, we found a significant reduction in patients only with a graph density corresponding to costs above 20 or below 10%.

Taken together, the results obtained with κ_*E*_*g*__, κ_*D*_, and κ_*B*_ indicate that a global reorganization is occurring in the contralesional hemisphere of patients. The results related to κ_*E*_*l*__ and κ_*C*_ suggest also a reorganization using metrics at the neighborhood level.

The comparison of the intra-hemispheric connectivity between patients and controls using the classical graph metrics are shown in the left column of the Figure 3. Global efficiency (*E*_*g*_), local efficiency (*E*_*l*_), betweenness centrality (*B*), and clustering (*C*) are displayed in the left column for cost values ranging between 5 and 30%. The only significant difference between both groups was found for *E*_*g*_ at 25 and 30% costs (Wilcoxon test, *p* < 0.05).

#### 3.2.2. κ per hemispheric lesion side

In order to explore whether our results could differ between left-sided and right-sided lesions, we computed the differences of κ between controls and patients at 20% cost, comparing left control hemispheres against left contralesional patients hemispheres (nine subjects) and right controls hemispheres against right contralesional patients hemispheres (11 subjects). In Figure 4A, we show the values of κ_*D*_ estimated for all the subjects, while in Figure 4B we plot the mean *D* of each node in the control group against the difference between groups in mean *D* of each node. We found a κ_*D*_ = −0.27 for the right-sided lesioned patients and κ_*D*_ = −0.36 for the left-sided lesioned patients. The cortical surface representation of the mean *D* differences between stroke patients and controls is shown in Figure 4C, where red denotes increased *D*, on average, in patients compared to controls while blue denotes abnormally decreased *D* in stroke patients. Finally, in Figure 4D, we show the brain regions that demonstrated significant between group differences in *D*, corrected for multiple comparison applying Wilcoxon test (*p* < 1/*N*) as indicated in Section 2.11. In right-sided stroke lesion, we found that the occipital cortex, which was high-*D* region in the normal brain networks, became a low-*D* region in the stroke brain networks. When the lesion is on the left side, we found the same trend in the lingual gyrus.

Similar results were obtained in the case of κ_*E*_*g*__ (see Figure S4), where κ_*E*_*g*__ = −0.35 for the right-sided lesioned patients and κ_*E*_*g*__ = −0.37 for the left-sided lesioned patients. We observed that calcarine area, cuneus and lingual gyrus, which were high-*E*_*g*_ regions in the normal brain networks, became low-*E*_*g*_ regions in the stroke brain networks (in both, left and right hemispheres), and also occipital lobe in left hemisphere; whereas the parietal inferior gyrus, which was low-*E*_*g*_ region in the normal group became high-*E*_*g*_ region in the patient group. Same analysis was performed on each subgroup of patients with κ_*E*_*l*__, κ_*B*_, and κ_*C*_. The corresponding results are displayed in the Supplementary Material in Figures S5–S7, respectively.

### 3.3. Robustness of the patients' results

We are concerned that the sample of patients is small, even smaller if we separate the patients into two subgroups, left and right sided lesions. In order to test if the significant differences found between patients and controls are robust, we performed a repeated bootstrap sampling from the HCP data.

We randomly selected 20 subjects that simulated the healthy controls, 11 subjects that played the role of left-sided lesion patients, and nine more subjects as right-sided lesion patients. For each random selection, we computed the κ of each subject and the *z*-values (Wilcoxon test) of the comparison of κ values between the control and patient groups for each graph metric. We repeated the same procedure 1000 times. The *p*-value was computed counting how many times the *z*-values were lower than the one we obtained with our true control and patient groups.

**Figure 5:**
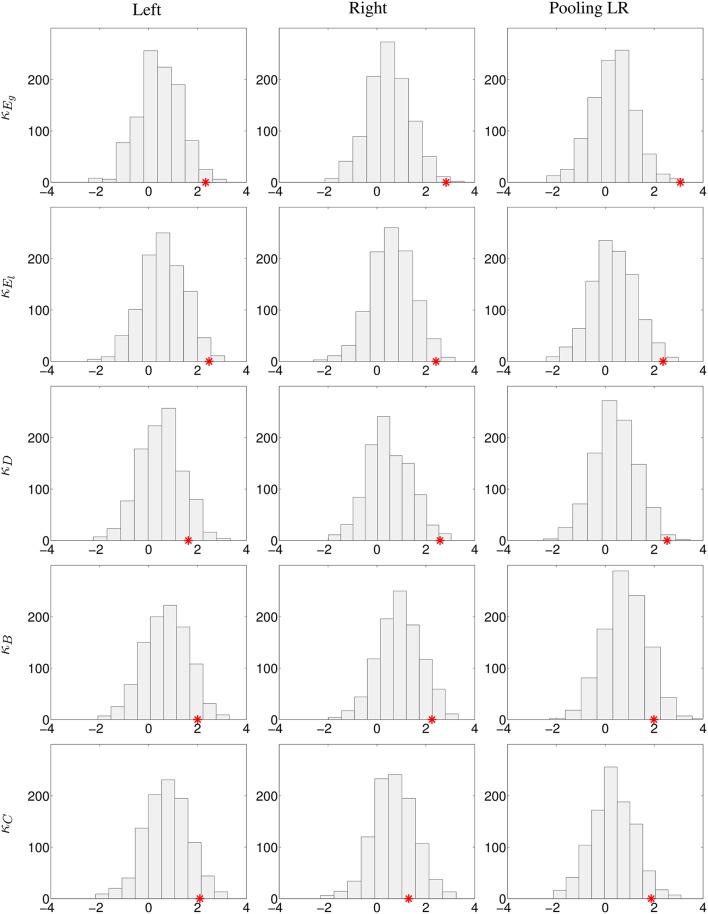
**Analysis of the robustness of the significant differences between controls and stroke patients using the HCP database**. 1000 bootstrap sampling iterations were performed. In each iteration, 40 subjects were randomly selected (20 subjects as healthy controls, 11 subjects as left-sided lesion patients, and 9 subjects as right-sided lesion patients), their respective κ index and the *z*-values (Wilcoxon test) of the comparison of κ between the control and patient groups for each graph metric were computed. Histograms with the bootstrap sampling of κ_*E*_*g*__, κ_*E*_*l*__, κ_*D*_, κ_*B*_, and κ_*C*_ at 20% cost are shown. First column, replication of left lesioned stroke; middle column, of right-sided lesion stroke; last column, pooling left and right-sided lesion groups, and compared against the mean between left and right hemispheres in controls. The red star corresponds to the true *z*-value obtained from the comparison of κ between patients and controls.

Two different experiments were performed: first, pooling left and right contralesional hemispheres (to test the results obtained in Figure 3 but only at 20% cost) and second, comparing left contralesional hemisphere in patients to left hemisphere in controls and right contralesional hemisphere in patients to right hemisphere in controls (to test the results obtained in Figure 4 and Figures S4–S7). Results are shown in Figure 5. With the limitation that our on-site data are not acquired in the same conditions than the HCP data, the bootstrap sampling tend to show that the results obtained on κ_*E*_*g*__ (left, *p* = 0.019; right, *p* = 0.005; pooling left and right, *p* = 0.003), κ_*E*_*l*__ (left, *p* = 0.016; right, *p* = 0.013; pooling left and right, *p* = 0.012) in the three cases and κ_*D*_ (left, *p* = 0.111; right, *p* = 0.011; pooling left and right, *p* = 0.006) in the left-sided lesion subgroup and pulling both sides lesions are significant. With κ_*B*_, there is only a trend of significance (left, *p* = 0.072; right, *p* = 0.075; pooling left and right, *p* = 0.096), while with κ_*C*_ (left, *p* = 0.073; right, *p* = 0.236; pooling left and right, *p* = 0.041), results are significant when pooling both sides lesions in the same group, and not significant dividing the patients into subgroups.

## 4. Discussion

In this study, we explored the “hub disruption index” (κ) that aims at capturing brains' networks reorganization in order to propose it as a new tool for clinical investigation of brain lesions.

### 4.1. Characteristics of κ: reliability, group discriminability

We first showed that κ is more reliable than global graph metrics in healthy subjects. We then applied it to explore the reorganization of the brains' contralesional hemispheric networks in the post-acute stage of severe stroke patients. We found significantly lower κ-values in the contralesional hemispheres of the patients' brain networks indicating the presence of reorganization in the contralesional hemisphere, a result that was not found when using classical graph metrics. Through this clinical example, we showed here that κ is more reliable than graph metrics and more sensitive to detect differences between groups of patients as compared to healthy controls.

κ index can be computed on different graph metrics. As shown in Figure 3, some κ metrics present higher group discriminability, as assessed by the significance of the group differences. κ appears to be more sensitive when computed on degree, global efficiency, and local efficiency. These results confirm those found with these classical metrics (Guo et al., 2012).

### 4.2. Sample size and group discriminability with κ

The ICC reliability relates to the variance of the measures. ICC is commonly classified into different categories (Cicchetti, 1994; Sampat et al., 2006): less than 0.4 indicates low reliability, 0.4 to 0.6 indicates fair reliability, 0.6 to 0.75 indicates good reliability, and greater than 0.75 indicates excellent reliability. However, there are several limitations of ICC approaches, as described by Müller and Büttner (1994). ICC estimation may vary according to the estimation method leading to different versions of ICCs and ICCs are dependent on the range of the measuring scale. Consequently, it has been recommended to calculate confidence intervals or *p*-values in addition to ICCs (Shrout and Fleiss, 1979).

Here, with a group of 20 subjects or higher, we showed that we can achieve reliable κ estimation for the whole brain connectivity analysis (*p* ~0.05) even if the ICC values are not very high. For the intra-hemispheric connectivity, κ estimation presents less reliability and thus a larger variance. As a consequence, in this case, the discriminability between two groups is more difficult to achieve, but when differences between groups are large enough, even small groups can be sufficient to detect the effect. This situation could be compared to the Student *t*-test: when the difference between two Gaussian curves is sufficient, this difference can be statistically significant even with large variance in the Gaussian curve and with a low number of degrees of freedom.

In this paper, the difference in κ between each sub-group and controls is so large that despite the small sample size, we could observe significant differences between both groups. In a recent study, the discriminability between groups was considered a criterion as important as the reliability for the purpose of translation to clinical studies and it was used in the evaluation of different connectivity methods, namely ROI-based analysis and ICA based analysis (Shirer et al., 2015). Here, we showed that graph based κ index is both reliable and has the ability to discriminate between groups.

### 4.3. κ as a measure of brain network reorganization

Since κ, for a given individual, is computed by linear regression of all nodal metric differences between this individual and the mean nodal metric computed on a group of controls (see Figure 1), the larger the differences between nodal metrics, the larger the κ. This index is thus specially sensitive to the combination of underconnected and overconnected brain regions, a situation that occurs in different neurological and psychiatric brain disorders in comparison to a control group. The significantly disconnected regions were found mainly in the occipital lobes and overconnected regions were in the superior parietal cortex. This pattern presents some similarities with the one found in the post-anoxic comatose patients (Achard et al., 2012). However, it should be noticed that resting state data were acquired with eyes open in controls, and that patients with severe subacute stroke were not all capable to keep their eyes opened during the whole session. Reorganization of the functional network in the eye-opened state compared to the eye-closed state has been reported in previous works, with decreased or increased efficiency at the nodes related to the default mode network and the visual network (Xu et al., 2014). Therefore, a question that remains to be addressed is how eye closure may have influenced the changes observed in the brain network.

### 4.4. Potential clinical interest of κ

The clinical interest of κ has been shown in different pathological conditions such as in disorders of consciousness (Achard et al., 2012), in epilepsy (Ridley et al., 2015), or in neuromyelytis optica (Hemmert et al., 2013). In comatose patients, we found that the brain connectivity was profoundly modified with both disconnected and overconnected nodes. κ was indeed deeply reduced in these patients as compared to healthy subjects (Achard et al., 2012).

At our knowledge, this is the first time that a global change of connectivity is observed within the contralesional hemisphere in stroke. In a computational model of focal brain lesions, Alstott et al. (2009) found that lesions produced specific patterns of altered functional connectivity among distant regions of cortex, often affecting both cortical hemispheres. In the clinical situation of reversible single hemisphere sedation, currently known as “Wada test,” that mimics single hemispheric lesions, large topological modifications affecting in particular the hubs of the networks were found with EEG investigation (Douw et al., 2009).

In many other brain disorders thought to be subtended by hubs lesions (Crossley et al., 2014), such as Alzheimer disease (Buckner et al., 2009), we argue here that this κ metric deserves to be used. However the relationship between κ and behavioral clinical scores remains to be explored to assess whether κ could be used as a surrogate biomarker.

### 4.5. Remaining issues about κ

Few methodological issues remain in the exploration of networks reorganization with κ. First, the variance within the reference group is not taken into account in the computation of κ. Second, the work done here was performed using the template AAL (Tzourio-Mazoyer et al., 2002) but few studies are using finer parcellation schemes with more reliable results (Termenon et al., 2016). Thus, the influence of the parcellation template needs to be explored in order to be able to choose the template providing the highest reliability together with a high group discriminability.

An other issue relates to the scan duration. Here, we considered the total scan duration available, corresponding to 14'24”. However, this duration is long and tiring for patients and a lot of clinical studies are acquired with shorter scan duration. It would thus be of interest to study the reliability of κ with respect to the scan duration. It is likely that κ presents a higher reliability as the scan duration increases, as shown in our previous study with the Human Connectome Project (Termenon et al., 2016).

The most challenging issue about κ is to interpret this metric in the context of brain networks. This could be addressed using different experiments on physiological parameters in animal models, for example. Such studies are out of the scope of this paper.

## Ethics statement

All patients provided written informed consent prior to their inclusion in the ISIS-HERMES study. In case of severe aphasia, written consent was provided by patient's relative. Furthermore, after recovery, an additional patient's informed consent was provided. The Ethics committee name is the CPP “Comité de protection des Personnes” Sud Est V. The CPP reference number of the Study is 07-CHUG-25. The AFSSAPS reference is 2007-A0083-50/5. The ClinicalTrial.gov identifier is NCT00875654.

## Author contributions

All authors contribute in this original research paper. The methodological design was performed by SA and MT, and the data computational work was performed by MT. The analysis of the results was done by SA, CD, and MT and the clinical contribution was done by AJ. All authors contributed in the manuscript: SA, CD, AJ, and MT. Finally, the acquisition of HERMES data was performed by AJ. All authors agree to be accountable for the content of the work.

## Funding

Allocation doctorale de Recherche de la région Rhône Alpes-Ref 13 009645 01.

### Conflict of interest statement

The authors declare that the research was conducted in the absence of any commercial or financial relationships that could be construed as a potential conflict of interest.

## Footnotes

^1^http://www.humanconnectome.org/

^2^https://clinicaltrials.gov/ct2/show/NCT00875654?term=ISIS+stroke+stem+cells&rank=1

^3^https://www.nitrc.org/projects/artifact_detect/

^4^http://cran.r-project.org/web/packages/brainwaver/index.html

^5^http://cran.r-project.org/web/packages/igraph/index.html
