# Supplementary material for: The “Hub Disruption Index,” a Reliable Index Sensitive to the Brain Networks Reorganization. A Study of the Contralesional Hemisphere in Stroke
Source: Front Comput Neurosci. 2016 Aug 17;10:84. doi: 10.3389/fncom.2016.00084 (PMC4987351; doi:10.3389/fncom.2016.00084)
Supplement: Supplementary file 1 [file DataSheet1.PDF]

Supplementary Material:
The ’Hub Disruption Index’, a reliable index sensitive to the brain networks reorganization. A study of the contralesional hemisphere in stroke

Maite Termenon *, **Sophie Achard, Assia Jaillard, and Chantal Delon-Martin**
*Correspondence:
Maite Termenon:
maite.termenon@ujf-grenoble.fr

Modified version of classical AAL parcellation scheme

The classical AAL parcellation scheme is composed by 116 regions including the cerebellum. We have merged some of the regions, reducing the parcellation to 89 regions. Merged regions are: frontal medial orbital and rectus (one region for left and one for right hemisphere); occipital superior, middle and inferior (one region for left and one for right hemisphere); temporal pole superior and medial (one region for left and one for right hemisphere); the cerebral crus (one region for left and one for right hemisphere); areas III, IV, V and VI of cerebellum (one region for left and one for right hemisphere); areas VII, VIII, IX, X of cerebellum (one region for left and one for right hemisphere) and finally, the vermis (one single region for both hemispheres).

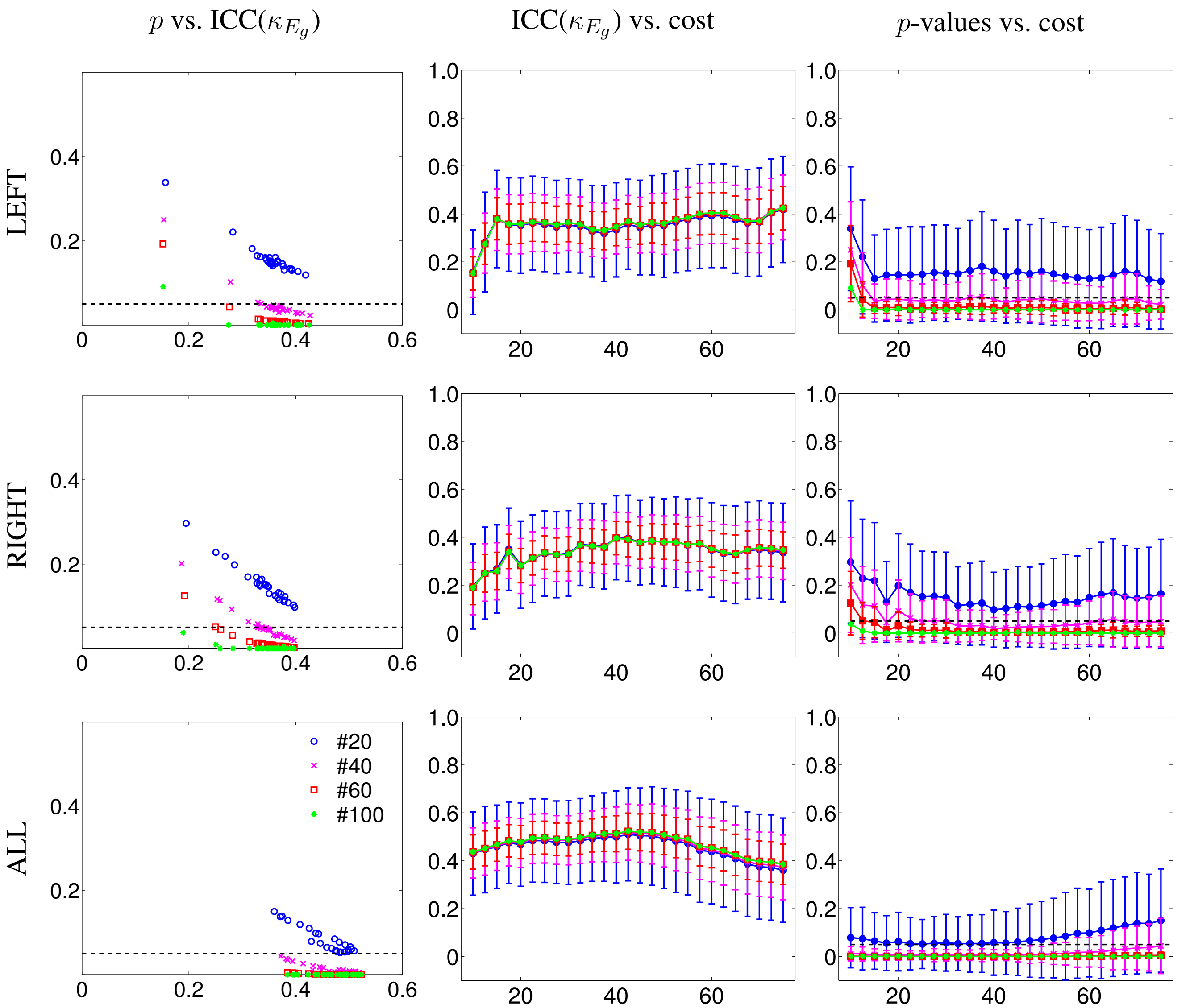

Figure S1. Reliability results for κ global efficiency (κ_{E_g}) in terms of number of subjects as a function of the cost from 10% to 75%, in steps of 2.5%. Results are given for subgroups of 20, 40, 60 and finally, 100 subjects using the database of the HCP project. First column, p-values of ICC (y-axis) as a function of ICC values (x-axis) for different number of subjects. Second column, values of ICC (y-axis) as a function of the cost (x-axis) for different number of subjects. Third column, ICC associated p-values (y-axis) as a function of the cost (x-axis) for different number of subjects. LEFT refers to the graph built from the left intra-hemispheric connections, RIGHT for the right intra-hemispheric connections. ALL refers to the graph built from connections of the whole brain. We found that κ_{E_g} is more reliable than mean global efficiency E_g (compare to Fig. S2)

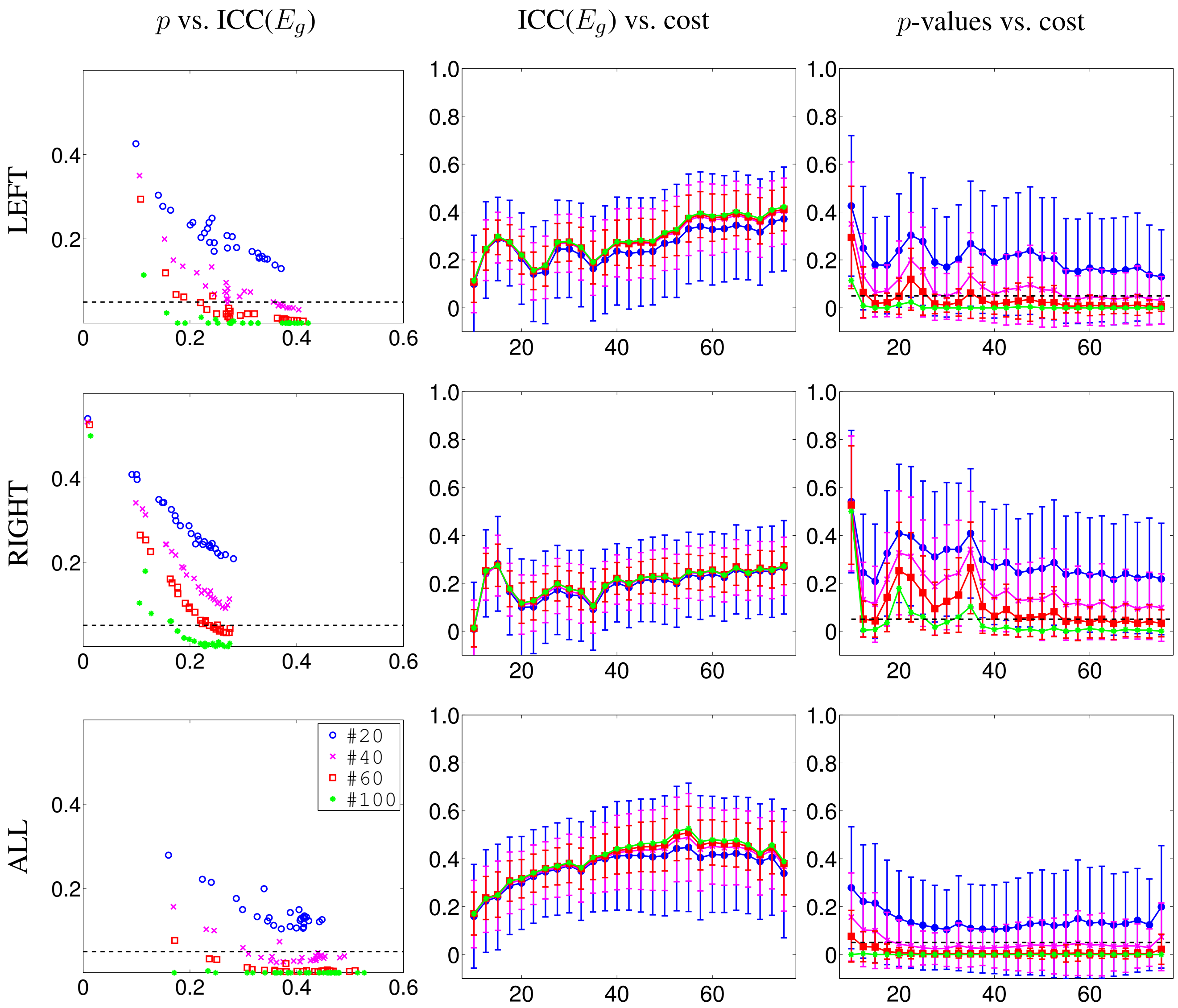

Figure S2. Reliability results for mean global efficiency (E_g) using the database of HCP project in terms of number of subjects as a function of the cost from 10% to 75%, in steps of 2.5%. Results are given for subgroups of 20, 40, 60 and finally, 100 subjects using the database of the HCP project. First column, p-values of ICC (y-axis) as a function of ICC values (x-axis) for different number of subjects. Second column, values of ICC (y-axis) as a function of the cost (x-axis) for different number of subjects. Third column, ICC associated p-values (y-axis) as a function of the cost (x-axis) for different number of subjects. LEFT refers to the graph built from the left intra-hemispheric connections, RIGHT for the right intra-hemispheric connections. ALL refers to the graph built from connections of the whole brain. We found that E_g is less reliable than κ global efficiency (κ_{E_g}) (compare to Fig. S1).

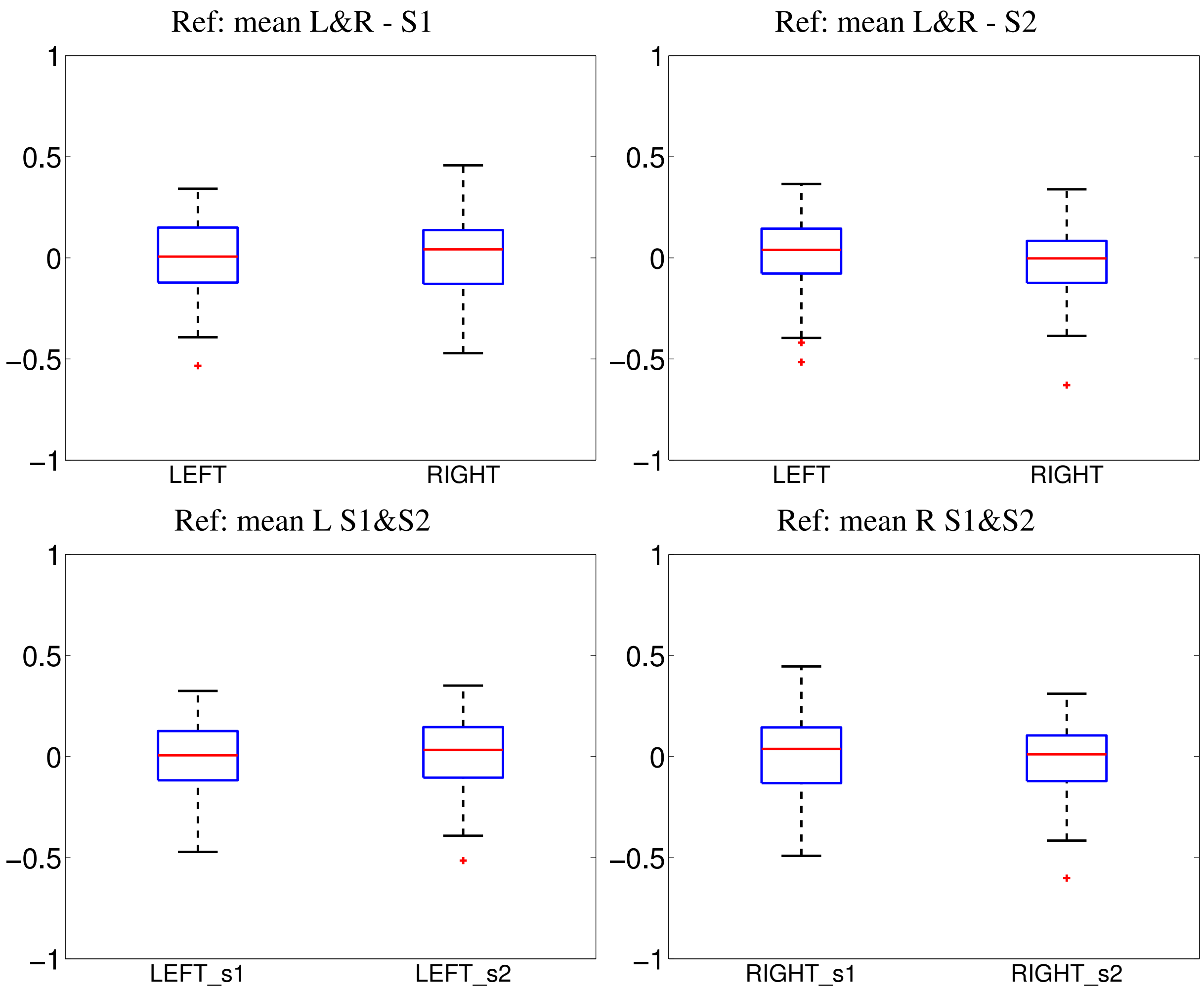

Figure S3. Boxplots of the differences between left and right hemispheres of κ degree (κ_D) considering the 100 healthy subjects together with their two sessions (S1 and S2) of the HCP database. First row shows the comparison between left and right hemispheres for each session independently. Second row shows the comparison between the two sessions of the left and right hemispheres separately, considering as reference the mean between both sessions of each hemisphere. Results show no significant differences in κ_D neither between left and right hemispheres, nor between different sessions of the same subjects.

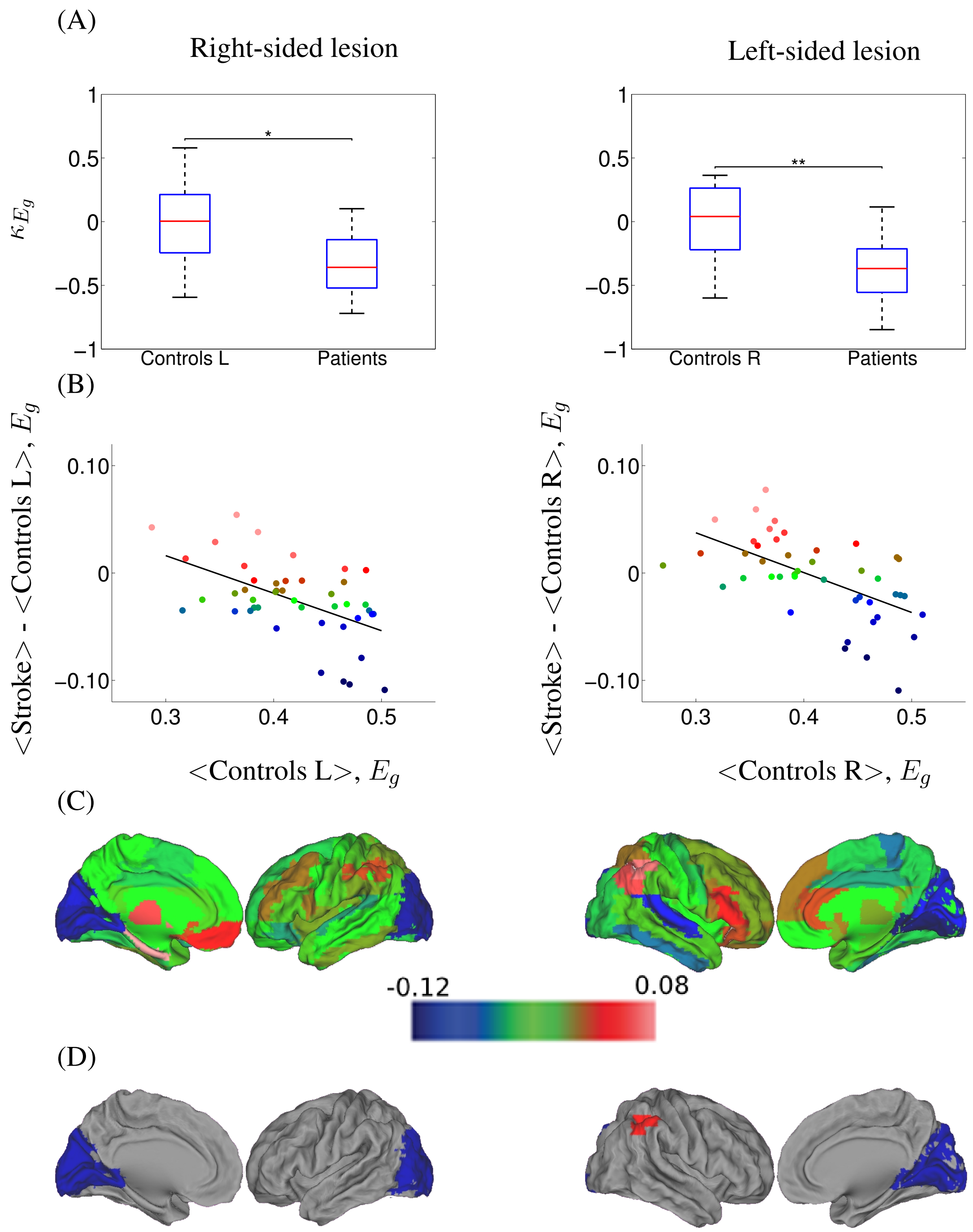

Figure S4. κ_{E_g} hub disruption of functional networks in stroke patients contralesional hemisphere, computed at a 20.0% cost. A: Boxplots of the individually estimated hub disruption indices for the healthy volunteer group and the stroke patient group. On the left, healthy volunteer group left hemisphere and stroke contralesional left hemisphere; on the right, healthy volunteer group right hemisphere and stroke contralesional right hemisphere. B: On the left, results of the healthy volunteer group left hemisphere and the stroke group with left contralesional hemisphere, where $\kappa = -0.35$; on the right, results of the healthy volunteer group right hemisphere and the stroke group with right contralesional hemisphere, where $\kappa = -0.37$. C: Cortical surface representation of the difference in mean E_g between both groups; red denotes increased E_g, on average, in patients compared with healthy volunteers; blue denotes abnormally decreased E_g in stroke patients. D: nodes that demonstrated significant between-group difference in nodal E_g; Wilcoxon test, $p < 0.023$; red denotes significantly increased E_g and blue denotes significantly decreased E_g in the patients on average.

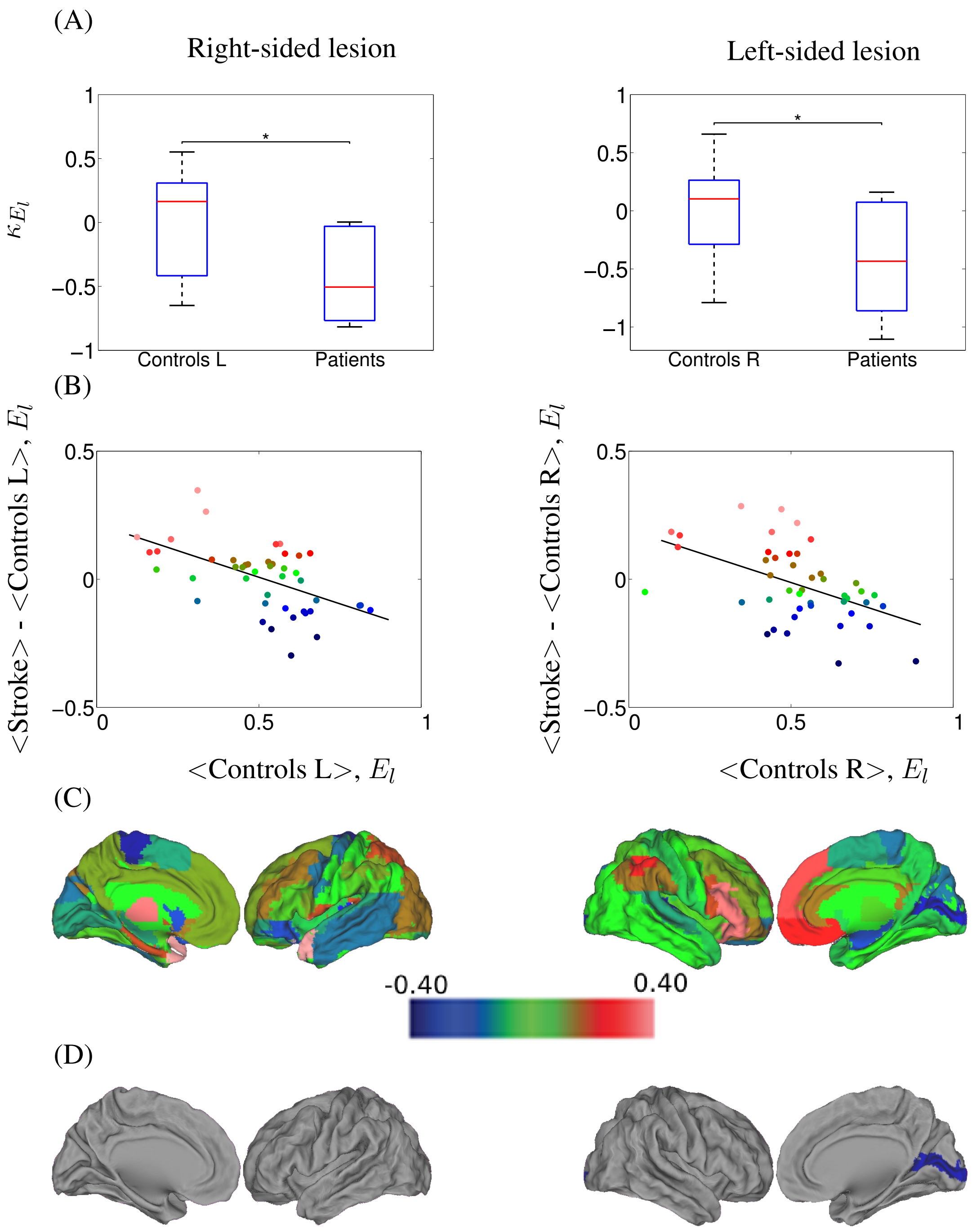

Figure S5. κ_{E_l} hub disruption of functional networks in stroke patients contralesional hemisphere, computed at a 20.0% cost. A: Boxplots of the individually estimated hub disruption indices for the healthy volunteer group and the stroke patient group. On the left, healthy volunteer group left hemisphere and stroke contralesional left hemisphere; on the right, healthy volunteer group right hemisphere and stroke contralesional right hemisphere. B: On the left, results of the healthy volunteer group left hemisphere and the stroke group with left contralesional hemisphere, where $\kappa = -0.41$; on the right, results of the healthy volunteer group right hemisphere and the stroke group with right contralesional hemisphere, where $\kappa = -0.41$. C: Cortical surface representation of the difference in mean E_l between both groups; red denotes increased E_l, on average, in patients compared with healthy volunteers; blue denotes abnormally decreased E_l in stroke patients. D: nodes that demonstrated significant between-group difference in nodal E_l; Wilcoxon test, $p < 0.023$; red denotes significantly increased E_l and blue denotes significantly decreased E_l in the patients on average.

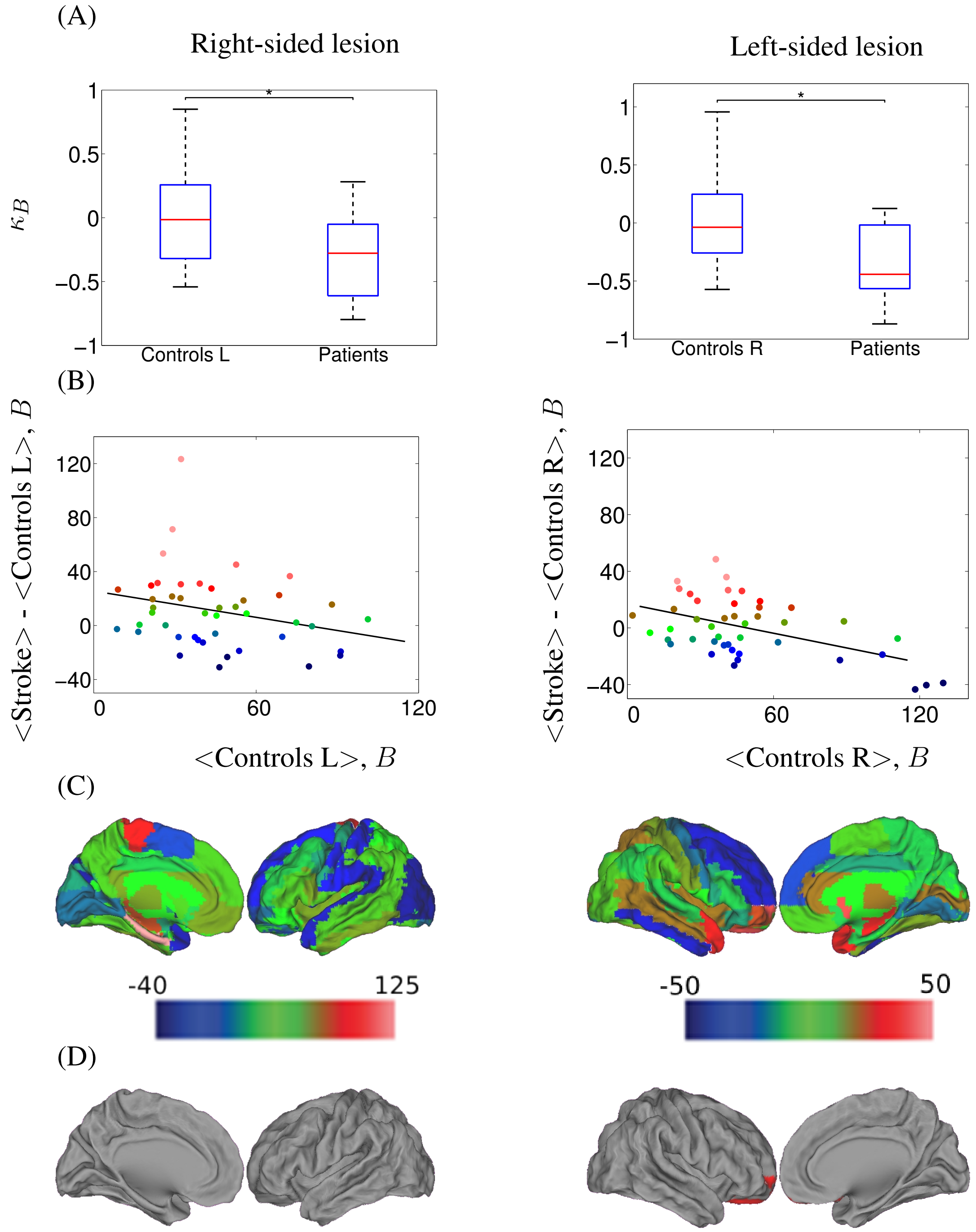

Figure S6. κ_B Hub disruption of functional networks in stroke patients contralesional hemisphere, computed at a 20.0% cost. A: On the left, results of the healthy volunteer group left hemisphere and the stroke group with left contralesional hemisphere, where $\kappa = -0.32$; on the right, results of the healthy volunteer group right hemisphere and the stroke group with right contralesional hemisphere, where $\kappa = -0.35$. B: Boxplots of the individually estimated hub disruption indices for the healthy volunteer group and the stroke patient group. On the left, healthy volunteer group left hemisphere and stroke contralesional left hemisphere; on the right, healthy volunteer group right hemisphere and stroke contralesional right hemisphere. C: Cortical surface representation of the difference in mean B between both groups; red denotes increased B, on average, in patients compared with healthy volunteers; blue denotes abnormally decreased B in stroke patients. D: nodes that demonstrated significant between-group difference in nodal B; Wilcoxon test, $p < 0.023$; red denotes significantly increased B and blue denotes significantly decreased B in the patients on average.

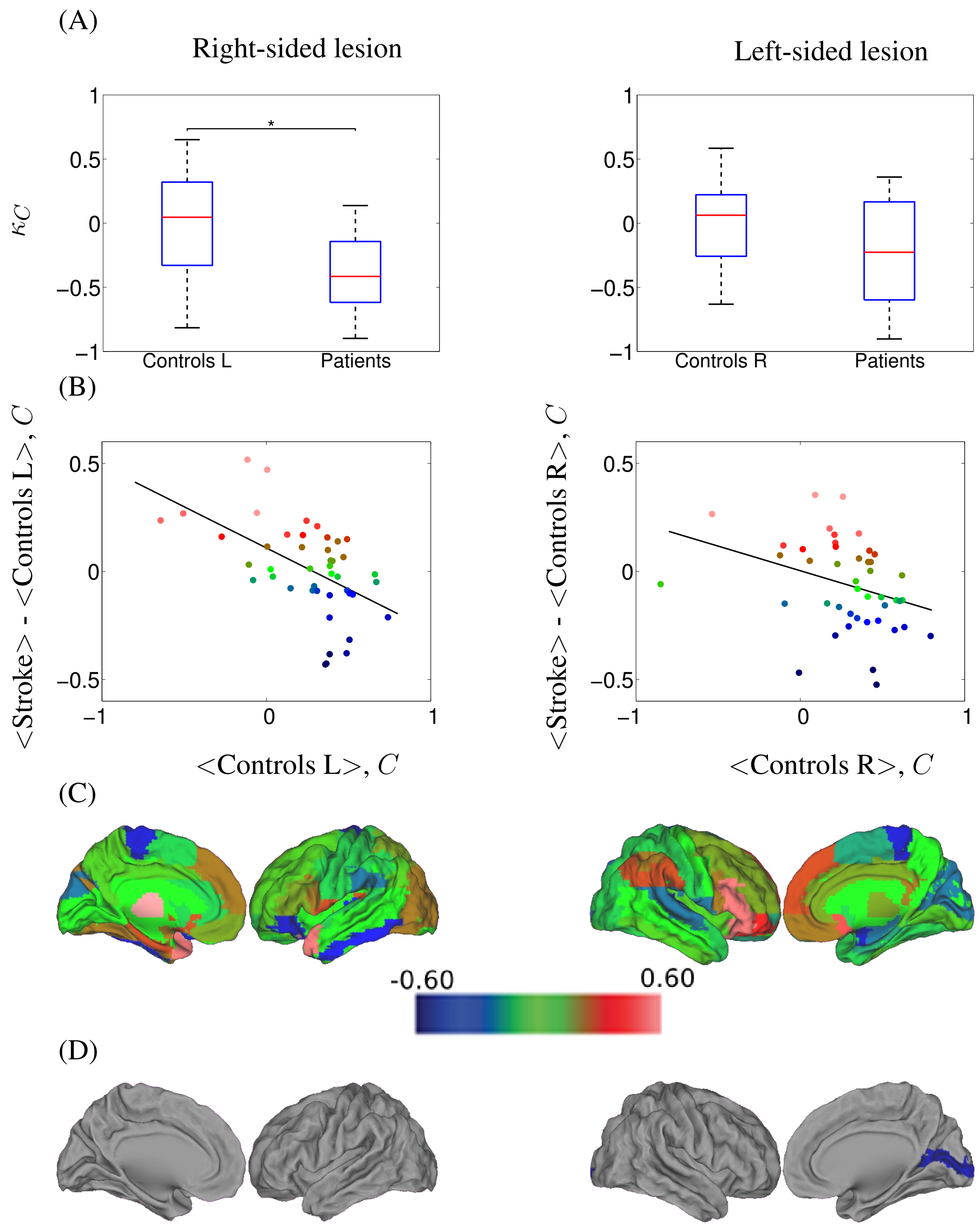

Figure S7. κ_C hub disruption of functional networks in stroke patients contralesional hemisphere, computed at a 20.0% cost. A: Boxplots of the individually estimated hub disruption indices for the healthy volunteer group and the stroke patient group. On the left, healthy volunteer group left hemisphere and stroke contralesional left hemisphere; on the right, healthy volunteer group right hemisphere and stroke contralesional right hemisphere. B: On the left, results of the healthy volunteer group left hemisphere and the stroke group with left contralesional hemisphere, where $\kappa = -0.38$; on the right, results of the healthy volunteer group right hemisphere and the stroke group with right contralesional hemisphere, where $\kappa = -0.23$. C: Cortical surface representation of the difference in mean C between both groups; red denotes increased C, on average, in patients compared with healthy volunteers; blue denotes abnormally decreased C in stroke patients. D: nodes that demonstrated significant between-group difference in nodal C; Wilcoxon test, $p < 0.023$; red denotes significantly increased C and blue denotes significantly decreased C in the patients on average.
